# Improved MRI detection of inflammation-induced changes in bone marrow microstructure in mice: a machine learning-enhanced T2 distribution analysis

**DOI:** 10.1186/s41747-025-00574-1

**Published:** 2025-03-26

**Authors:** Luise Brock, Hadas Ben-Atya, Ashish Tiwari, Dareen Saab, Narmeen Haj, Lukas Folle, Galit Saar, Andreas Maier, Moti Freiman, Katrien Vandoorne

**Affiliations:** 1 Faculty of Biomedical Engineering, Technion-Israel Institute of Technology, Haifa, Israel; 2 Department Informatik, Friedrich-Alexander-Universität Erlangen-Nürnberg, Erlangen, Germany; 3 Biomedical Core Facility, Rappaport Faculty of Medicine, Technion-Israel Institute of Technology, Haifa, Israel

**Keywords:** Bone marrow diseases, Deep learning, Inflammation, Mice (inbred C57BL), Magnetic resonance imaging

## Abstract

**Background:**

We investigated inflammation-induced changes in femoral hematopoietic bone marrow using advanced magnetic resonance imaging (MRI) techniques, including T2-weighted imaging, scalar T2 mapping, and machine learning-enhanced T2 distribution analysis to improve the detection of bone marrow microstructural alterations. Findings were correlated with histological markers and systemic inflammation.

**Methods:**

Using a 9.4-T magnet, T2-weighted and multislice multiecho sequences were applied to evaluate bone marrow in female C57BL/6J mice divided into three groups: (1) controls; (2) lipopolysaccharide-induced acute inflammation (LPS); and (3) streptozotocin (STZ)- and LPS-induced diabetic inflammation (STZ + LPS). T2 relaxation times and their distributions with scalar mapping and model-informed machine learning (MIML) were analyzed. Correlations with histological iron levels and blood neutrophil counts were assessed.

**Results:**

T2-weighted imaging showed a reduced signal-to-noise ratio in inflamed bone marrow (*p* = 0.034). Scalar T2 mapping identified decreased T2 relaxation times (*p* = 0.042), moderately correlating with neutrophil counts (*ρ* = 0.027) and iron levels (ρ = 0.016). MIML-enhanced T2 distribution analysis exhibited superior sensitivity than scalar T2 mapping, revealing significant reductions in the first T2 distribution peak (*p* = 0.0025), which strongly correlated with neutrophil counts (*ρ* = 0.0016) and iron sequestration (*ρ* = 0.0002). Histology confirmed elevated iron deposits in inflamed marrow, aligning with systemic inflammation.

**Conclusion:**

Combining T2-weighted imaging, scalar T2 mapping, and MIML-enhanced T2 distribution analysis offers complementary insights into inflammation-induced bone marrow remodeling. T2 distribution analysis emerged as a more sensitive tool for detecting microstructural changes, such as iron sequestration, supporting its potential as a noninvasive biomarker for diagnosing and monitoring inflammatory diseases.

**Relevance statement:**

This study highlights the potential of advanced MRI T2 analysis and machine learning methods for noninvasive detection of inflammation-induced microstructural changes in bone marrow, offering promising diagnostic tools for inflammatory diseases.

**Key Points:**

- This study investigated inflammation-induced changes in bone marrow with T2 MRI and MIML.
- MIML outperformed quantitative scalar T2 analysis, increasingly detecting inflammation and iron sequestration in the hematopoietic bone marrow.
- T2 MRI with MIML analysis could aid in the early diagnosis and management of inflammatory diseases.

**Graphical Abstract:**

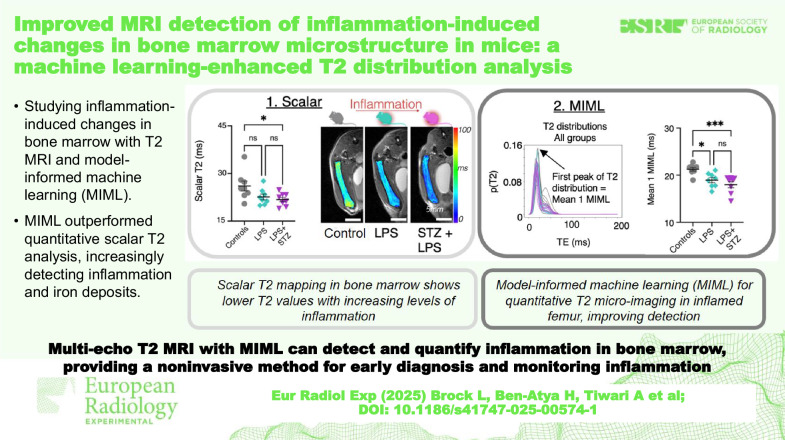

## Background

Globally, over 50% of deaths in humans are linked to inflammatory diseases [1]. Conditions like sepsis, diabetes, cancer, and neurodegenerative disorders progress through the activation of the immune system by inflammatory pathways [1]. In sepsis, where the immune response to infection is unleashed, elevated levels of circulating innate immune cells significantly correlate with higher mortality rates [2]. Diabetic individuals, who often experience chronic inflammation, face an even greater risk of severe sepsis outcomes than nondiabetic individuals [3].

A key part of inflammation involves the activation of innate immune cells—monocytes, macrophages, and neutrophils—which originate from hematopoietic stem and progenitor cells in the adult bone marrow [4]. These stem cells give rise to all blood cell types, including myeloid cells, which function as the innate immune system’s frontline defense against pathogens [5–7]. On average, the human bone hematopoietic marrow produces around 280 billion hematopoietic cells daily [8], a rate that increases significantly in response to systemic inflammation [9–12]. During inflammation, the bone marrow rapidly releases neutrophils and monocytes into the peripheral blood, prompting structural and functional changes within the hematopoietic bone marrow environment as cells are mobilized and replenished [10, 11, 13, 14]. While the diaphysis of long bones, such as the femur, undergoes conversion to fatty (yellow) marrow with age [15], the metaphyseal region near the growth plate retains hematopoietically active red marrow, even in adults [16]. Since inflammation is a major driver of chronic diseases, developing a universal biomarker for inflammation by probing hematopoietic activity in the bone marrow could provide a more comprehensive and dynamic view of immune system function.

Noninvasive imaging holds significant potential for identifying and tracking complex changes within the hematopoietic bone marrow. Fluorodeoxyglucose positron emission tomography (^18^F-FDG PET) and functional magnetic resonance (MRI) techniques, such as diffusion-weighted imaging (DWI), proton density fat fraction, and R2 or R2^*^ mapping, have been used to evaluate bone marrow abnormalities in lymphoma, myelofibrosis, and multiple myeloma [17–24]. ^18^F-FDG PET detects glycolytic activity [23, 24], DWI measures cellular density [17, 18], proton density fat fraction quantifies fat content [20], and R2 and R2^*^ assess iron deposition [13, 19]. Our recent work highlights significant bone marrow changes during inflammation using tracer-based imaging [10, 11, 13]. The integration of PET and MRI allows for simultaneous whole-body imaging, offering comprehensive insights into bone marrow pathologies. However, PET implies ionizing radiation exposure, which poses certain risks. In contrast, MRI presents a safer alternative, providing noninvasive, contrast-agent-free imaging that enhances efficiency without any ionizing radiation exposure.

Conventional scalar T2 mapping, a multiecho MRI method, provides macroscopic insights by fitting an exponential decay to T2-weighted images acquired at multiple echo times (TEs). However, its correlation with age [25] limits its utility in differentiating inflammation levels. Unlike scalar T2 mapping, which yields a single scalar value per pixel, T2 distribution unveils a spectrum of decay behaviors within each pixel. Using multiple images typically acquired with a multislice multiecho sequence, T2 distribution analysis fits multiple exponentially decaying components [26]. Raj et al [27], demonstrated the efficacy of Gaussian distributions in modeling T2 distributions, enabling a more nuanced characterization of tissue microstructure.

Recent advancements in deep neural network (DNN)-based techniques have further expanded the utility of T2 analysis [28, 29]. These methods enable the calculation of relative tissue compartment contributions, offering a detailed analysis of structural composition [28, 29]. T2 distribution analysis provides a robust method for characterizing tissue microstructure, overcoming the limitations of single-component T2 relaxometry by incorporating heterogeneity and partial volume effects. A significant advancement in this area is the model-informed machine learning (MIML) approach, which integrates MRI data with machine learning models to achieve detailed tissue composition analysis. MIML leverages prior knowledge and data-driven insights to disentangle complex tissue properties, enabling accurate estimation of microscopic composition. This technique has been successfully applied in brain imaging to enhance the interpretation of T2 data, offering precise differentiation of tissue compartments and structures [28].

Our study aimed to improve MRI T2-based detection of inflammation-induced changes in bone marrow microstructure using advanced T2 distribution analysis coupled with MIML. Specifically, we aimed to characterize structural and functional changes in femoral bone marrow during varying levels of inflammation using multi-echo T2 MRI, establish correlations between scalar T2 values, T2 distribution metrics, and inflammatory markers (such as neutrophil levels and histological iron deposits), and explore the impact of acute and diabetic inflammation in mouse models. By applying MIML, we seek to extract detailed T2 distributions that provide nuanced insights into bone marrow microstructure and demonstrate superior sensitivity compared to conventional scalar T2 mapping. Validation through histological analysis, including quantification of iron levels with Prussian blue staining, will further corroborate MRI findings. Ultimately, this research aims to support the development of targeted therapies to mitigate bone marrow hematopoietic activation during inflammation, while advancing safe and efficient imaging solutions with translational potential.

## Methods

### Animals

**Fig. 1:**
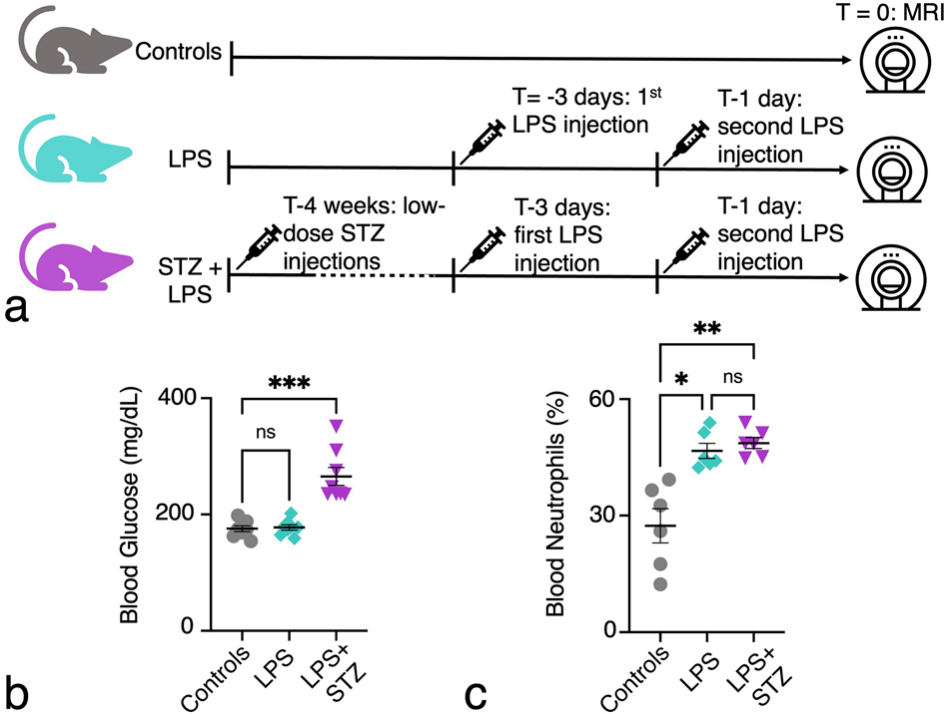
*In vivo* setup evaluating the bone marrow of mice with increasing inflammatory status by high-field 9.4-T magnetic resonance imaging (MRI). **a** (1) Healthy mice served as our control group (controls). (2) Mice were given LPS in their muscle to create a moderate level of inflammation for three days and then one day before MRI (LPS). (3) Diabetic mice, induced by STZ four weeks prior, subsequently received two intramuscular injections of LPS (STZ + LPS), leading to the highest level of inflammation compared to the other experimental groups. **b** Verification of glucose levels before injection of LPS in all of the experimental groups. **c** Neutrophil levels in all groups of mice at the end of the experiment

This study involved 24 female C57BL/6J mice, obtained at 8 weeks of age from Envigo (Israel) and imaged at 12 weeks. Acute inflammation was induced using lipopolysaccharide (LPS), a toll-like receptor 4 ligand that activates the innate immune response, increasing pro-inflammatory cytokines and neutrophil levels [11, 14]. Streptozotocin (STZ) was used to destroy pancreatic beta cells, inducing hyperglycemia and mimicking diabetic inflammation [13]. To model heightened inflammation, LPS and STZ were combined [13]. Mice were randomly assigned to three groups (*n* = 8 per group) to assess inflammation levels (Fig. 1a):

1. control healthy mice (controls);
2. mice receiving two intramuscular injections of LPS (100 ng from *Escherichia* *coli* O55:B5, Sigma-Aldrich, Darmstadt, Germany) in the left vastus lateralis muscle, administered three days and one day prior to imaging (LPS); and
3. diabetic mice induced with five low-dose STZ injections (50 mg/kg in NaCitrate, Sigma-Aldrich) four weeks prior, followed by LPS injections as above (STZ + LPS) [13].

Glucose levels were tracked in diabetic mice (Fig. 1b). After imaging, femurs were collected for histology, and blood was withdrawn from the heart to analyze blood counts using a hematology analyzer (ProCyte Dx, IDEXX Laboratories, Westbrook, ME, USA) to analyze neutrophil levels. All mice reached the endpoint, with no dropouts due to diabetes or other complications. All animal procedures, including handling, care, and experimentation, were conducted in compliance with the guidelines approved by the Technion Institutional Animal Care and Use Committee.

### Preclinical MRI

**Fig. 2:**
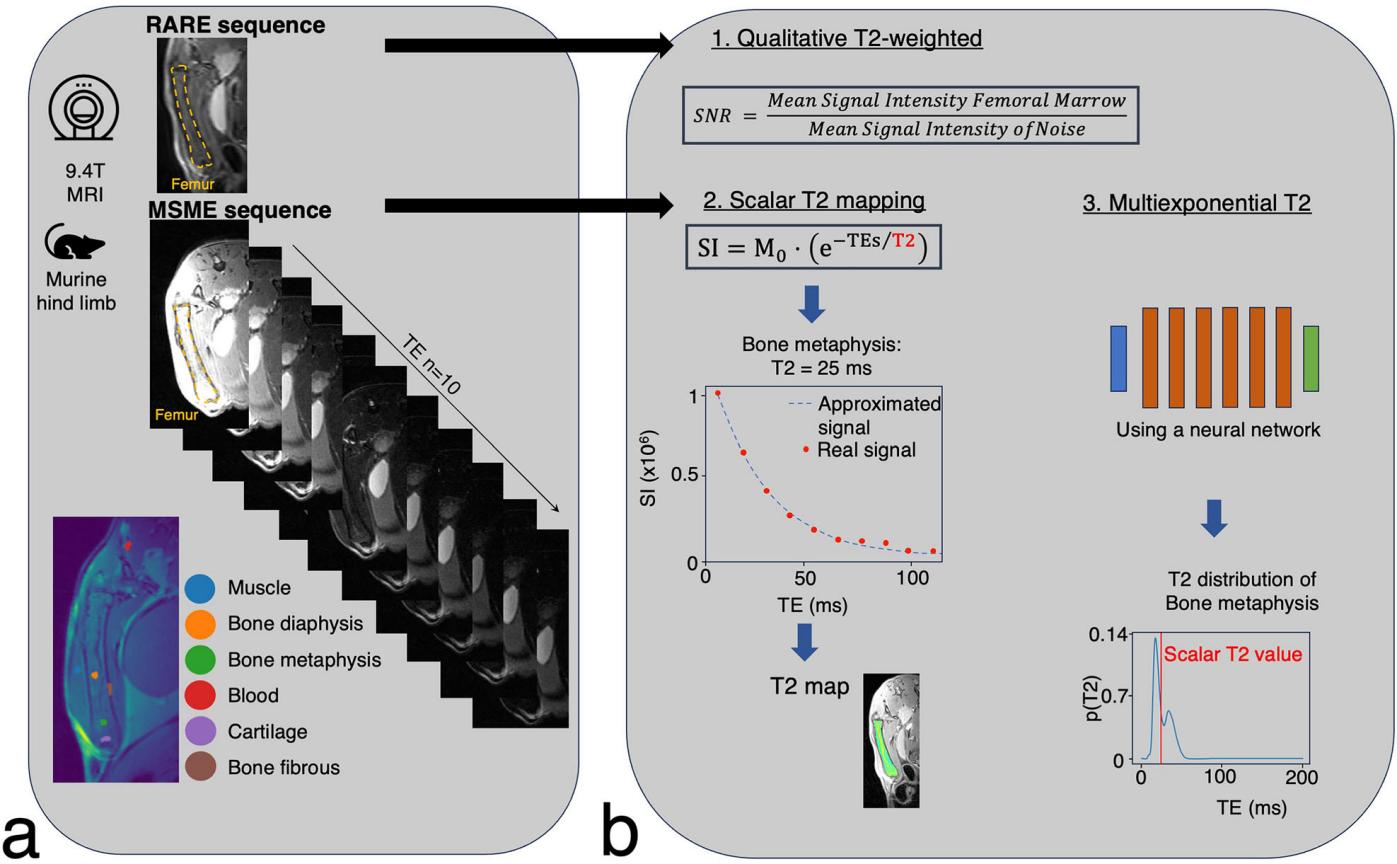
Overview of MR data acquisition and image analysis for T2-weighted imaging, scalar T2 mapping, and multiexponential T2 distribution. **a** Images of the mouse hind limb were acquired using a ‘Rapid acquisitions with refocused echoes’—RARE sequence for qualitative T2-weighted imaging and a multislice multiecho (MSME) sequence on a 9.4-T preclinical magnet for quantitative T2 mapping. Regions of interest (ROIs) were marked in distinct colors for analysis. The ROI of the cartilage was taken at the growth plate (purple). **b** The acquired data were analyzed using qualitative T2-weighted signal-to-noise ratio (SNR) measurements and quantitative approaches, including scalar T2 mapping and multiexponential T2 distribution analysis, to provide both qualitative and quantitative insights into bone marrow properties

Murine MRI images of the left limb were acquired using a 9.4-T horizontal bore MRI system (Bruker Biospec; Ettlingen, Germany), with an 86-mm volume coil for transmission and a 20-mm surface coil for detection. Animals were anesthetized (3.5% induction; 1.25% maintenance in 0.7 L/min oxygen), positioned on their right side while and kept warm using circulating hot water. Respiration was monitored and maintained at 40–80 bpm (Small Animal Instruments, Stony Brook, NY, USA). T2-weighted images were obtained using a rapid acquisition with refocused echoes (RARE) sequence with: TE = 40 ms, repetition time (TR) = 4,000 ms, echo spacing = 10 ms, RARE factor = 10, 8 excitations, FOV = 40 × 40 mm^2^, matrix = 256 × 256, slice thickness = 1 mm, 20 slices, and a 12:20 min scan time. A multislice multiecho sequence with fat suppression covering the left femur was used to acquire a series of T2-weighted images (Fig. 2a) [26, 30] was performed with: TR = 2 s, TEs = 10.4–103.9 ms, three slices (0.7 mm thick), FOV = 4 × 4 cm, matrix = 400 × 400, in-plane resolution = 100 × 100 µm², two excitations, and a 13:20 min scan time.

### Region-of-interest (ROI)

Images were preprocessed using ITK Snap 3.8 [31] (Philadelphia, PA, USA) for visualization and ROI annotation, focusing on the bone metaphysis as the primary site of hematopoietic stem and progenitor cells, key drivers of leukocyte production during inflammation [10, 11, 13]. While emphasizing the metaphysis, we aimed to include all bone marrow tissue components in the analysis.

### T2-weighted and scalar T2 analysis

T2-weighted imaging was used to qualitatively visualize tissue differences, while scalar T2 mapping provided quantitative measurements of T2 relaxation times [32]. Signal-to-noise ratio (SNR) was calculated by comparing femoral bone marrow ROI signal intensity to background noise outside the animal:

$${{{\rm{SNR}}}}=\,\frac{{{{\rm{Mean}}}}\;{{{\rm{signal}}}}\;{{{\rm{intensity}}}}\;{{{\rm{femoral}}}}\;{{{\rm{marrow}}}}}{{{{\rm{Mean}}}}\;{{{\rm{signal}}}}\;{{{\rm{intensity}}}}\;{{{\rm{of}}}}\;{{{\rm{noise}}}}}$$

Scalar T2 analysis involved drawing ROIs on grayscale images, followed by pixel-wise fitting using custom Matlab R2022b (Mathworks, Natick, MA, USA) software via Python. T2 relaxation times were quantified using:

$${{{\rm{SI}}}}\,({{{\rm{TE}}}})={{{\rm{SI}}}}_{0}\;\times\;{e}^{(-1/T2\,x\,{{{\rm{TE}}}})}\;+\;C$$

is the signal at TE = 0 ms, T2 is the transverse relaxation time, and *C* is the noise at the longest TE. Scalar T2 mapping was compared to T2 distribution analysis for detailed tissue characterization [30, 33].

### T2 distributions

The MIML network, used in this study, consists of six hidden layers with 256 neurons each, employing Mean Squared Error Loss and Wasserstein-Distance as loss functions [34, 35]. Originally designed for brain MRI data, it was adapted here for murine femoral imaging, taking normalized signals from ten echoes and outputting T2 distributions (p(T2)) on a logarithmic scale (0–200 ms). Simulated datasets (400,000 signals per compartment) based on known tissue parameters of each compartment (mean, SD) facilitated efficient, ethical model training of the MIML-based neural network [36].

Following MIML methodology [21], T2 distributions were modeled as Gaussian mixtures with compartment fractions sampled from a Dirichlet distribution, while the mean and standard deviation were sampled from a uniform distribution with the ranges described in Table 1. MRI signal simulations were based on extended phase graph algorithms [37], incorporating TE, flip angles, and SNR variations [38]. Furthermore, the tissue-specific T2 distribution is included in the creation of simulated signals, for which the compartment fraction, mean, and standard deviation are needed. MIML methodology [21] ensured the robustness of T2 distribution estimation across varying noise levels. The realistic SNR was measured using the first echo, and during training, we randomly varied the SNRs of the signals by introducing random noise to encompass the potential SNR range of the voxels. Noise robustness was ensured using Rician noise, with 20% of simulated data reserved for validation.

**Table 1:** T2 relaxation time parameters for various tissues

Tissue	Mean T2 range (ms)	Standard deviation range (ms)
Bone fibrous	10–20	2–5
Cartilage	35–60	6–9
Blood	170–200	10–15
Red bone marrow	30–45	6–9
Yellow bone marrow	15–25	2.5–4.5

These parameters have been utilized in the simulated signal generation using extended phase graph modeling, accurately representing the tissues of interest within our dataset

To achieve accurate estimation of T2 distributions of images of murine femoral bone marrow, PyTorch 1.13, a popular open-source machine learning library, implemented in Python 3.8 was used. The models were trained and evaluated on an NVIDIA Tesla graphics card using CUDA 11.3, a parallel computing platform used for GPU acceleration. The epoch with the lowest validation loss was selected as the final model. T2 distribution data from the brain [28, 29], were adapted for use in our 9.4-T MRI setup. These values, derived from the brain [39], spleen [40], and tumor [41] research, informed signal simulations for bone marrow compartments compartments (*e.g*., blood, yellow/red marrow, fibrous tissue, cartilage) (Table 1) [42]. The simulated signals were generated to reflect realistic combinations of tissues commonly found in bone marrow. Common combinations include blood and bone fibrous, blood and yellow bone marrow, blood and red bone marrow, red bone marrow and yellow bone marrow, bone fibrous and yellow bone marrow, and bone fibrous and red bone marrow. Subsequently, images underwent analysis with the trained MIML model; this process enabled the estimation of new parameters from the T2 distributions specific to regions within the MR images.

Gaussian mixture models (GMM) were applied to extract means and standard deviations from T2 distributions.

ROIs were annotated in the metaphysis and diaphysis to quantify red and yellow marrow, respectively [43]. In mice, the growth plates do not fuse after sexual maturation [44]. After annotating the ROIs, the T2 distributions were generated from these small, annotated regions of about nine pixels using the previously trained neural network (Fig. 2b), with results compared to scalar T2 mapping.

To visualize differences between the T2 distributions that aren’t visible a first look, the GMM post-processing method identified the means, variances, and weights of the different peaks in each distribution [45]. This model operates by maximizing the likelihood of the observed data, essentially determining the set of parameters that maximizes the probability of observing the data given the assumed Gaussian mixture. Depending on how many peaks are visible in the distribution, the GMM fits mostly between one and three Gaussian curves to the distribution. The parameters that describe the distribution are then plotted according to groups and regions so that differences can be visualized.

### Histology

To compare MRI findings with ground truth, histological exams were performed on the left femur bone marrow. After MRI, animals were euthanized, femurs isolated, decalcified for 4 days using MoL Decalcifier (ethylenediaminetetraacetic acid-based decalcifying solution; Milestone, Bergamo, Italy), and paraffinized. Section (4 µm thick) were cut, mounted, and stained with Perls’ Prussian blue for iron, appearing as distinct blue deposits. Slides were scanned under the light microscope (Slide scanner 250 Flash III, 3D Histech Ltd, Budapest, Hungary) and analyzed using Fiji (NIH, Bethesda, MA, USA).

### Statistical analysis and microscopic validation

The data is represented as means ± standard error of the mean, as it reflects the confidence in the reported means rather than data variability. Statistical analyses were performed with Prism 10 (GraphPad Software, San Diego, CA, USA) and using Python. First, normal distribution tests were carried out (d’Agostino and Pearson test) and all data failed the normal distribution test. Therefore, we further used non-parametric Kruskal–Wallis with a Dunn’s *post-hoc* test for comparison among the three groups. Nonparametric Spearman correlation analysis was performed to correlate scalar T2, T2 distribution, iron count, and blood neutrophils with each other. A *p*-value lower than 0.05 was considered significant. Statistical tests and animal numbers for each graph are specified in the figure legends.

## Results

**Fig. 3:**
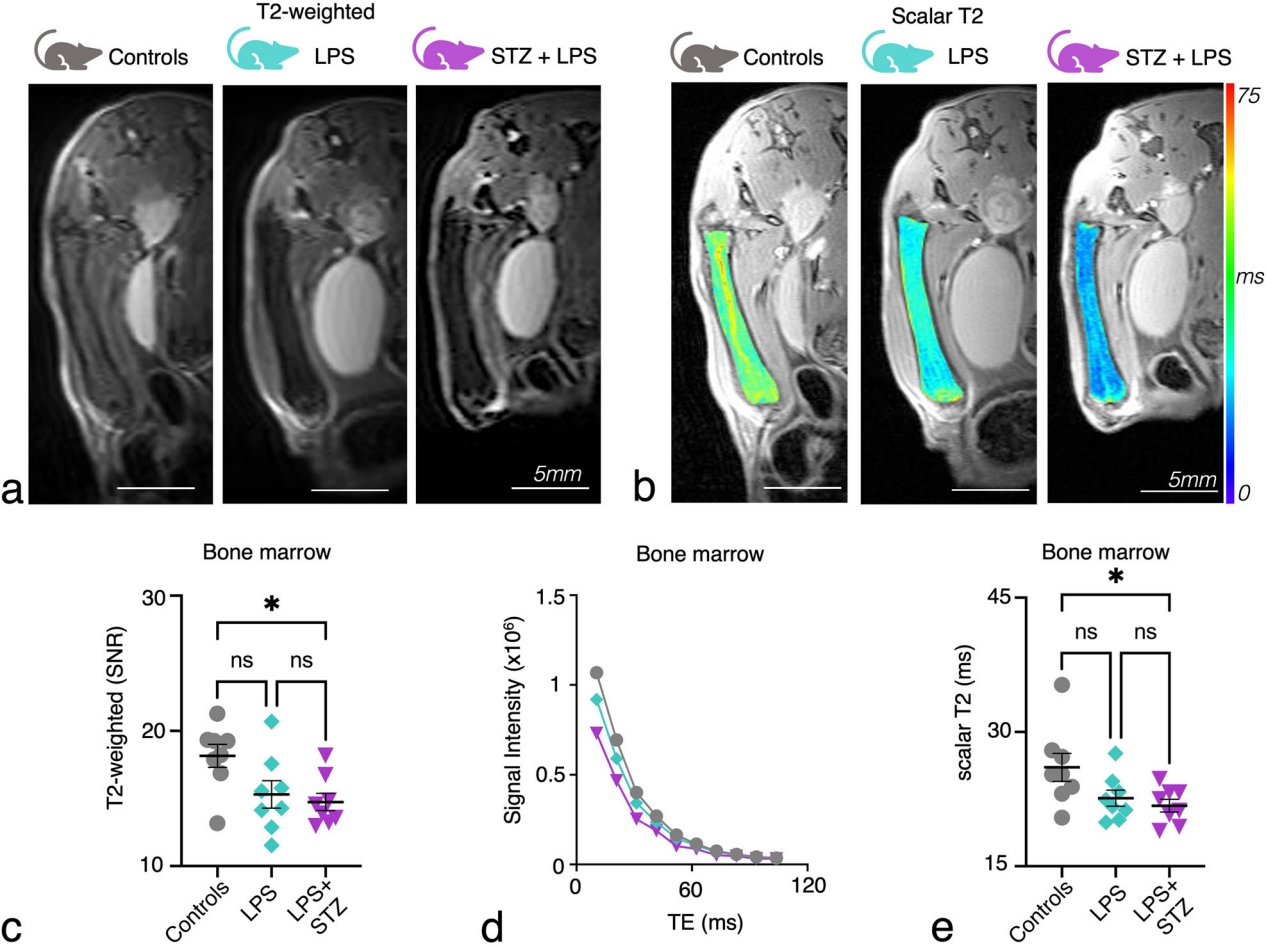
T2-weighted and scalar T2 mapping shows lower T2 values in the femoral bone marrow during inflammation. **a** Representative MR images using T2-weighted RARE of femoral bone marrow with increasing levels of inflammation. **b** Representative images with an overlay of a T2 map of femoral bone marrow (using a multislice multiecho sequence with variable TEs). **c** Signal-to-noise values (SNR) of femoral bone marrow on qualitative T2-weighted images among the experimental groups. **d** T2 decay curves for femoral metaphysis in each of the groups. **e** Scalar T2 values for the femoral metaphysis (controls: a control group of healthy mice; LPS: lipopolysaccharide-injected mice (acute inflammation model); STZ + LPS: STZ-induced diabetic mice with subsequent LPS injections (diabetic and acute inflammation model). Each dot represents one mouse (*n* = 8 mice per group. Kruskal–Wallis test: *p < 0.05; ns, Not significant; TE, Echo time

Inflammation alters hematopoiesis, increasing leukocyte production and reshaping bone marrow composition, with the femoral metaphysis as a relevant target site for assessing these changes [10, 11, 13]. To explore how inflammation affects bone marrow microstructure, we focused on evaluating T2 relaxation times using T2-weighted imaging and quantitative T2 mapping. This approach aims to link changes in bone marrow T2 values with systemic inflammatory responses, providing insight into the potential of T2 mapping as a noninvasive marker of bone marrow inflammation. Qualitative analysis of T2-weighted RARE’ images revealed a slight but statistically significant reduction in SNR values among the experimental groups, as shown in Fig. 3a and Table 2. Statistical significance was confirmed through Kruskal–Wallis analysis, with significant pairwise comparisons evident in Dunn’s *post-hoc* tests for controls *versus* LPS and controls *versus* STZ + LPS, while no significant difference was observed between LPS and STZ + LPS groups (Fig. 3c and Table 2). T2 maps and T2 decay curves of the bone marrow demonstrated a subtle decrease in T2 values with escalating inflammation (Fig. 3b, d). Quantitative analysis revealed a modest yet statistically significant reduction in scalar T2 values across the experimental groups (Fig. 3e and Table 2). Statistical significance was confirmed through Kruskal–Wallis analysis, with significant pairwise differences for controls *versus* LPS and controls *versus* STZ + LPS, while no significant difference was observed between LPS and STZ + LPS groups (Fig. 3e and Table 2). These findings underscore the utility of both qualitative and quantitative MRI techniques in detecting subtle tissue alterations associated with inflammation.

**Table 2:** Obtained *p*-values for statistical tests of T2 analyses in bone metaphysis

Test	T2-weighted	Scalar T2	Model-informed machine learning mean 1
Kruskal–Wallis	0.034	0.042	0.0025
Dunn: controls *versus* LPS	0.121	0.198	0.0362
Dunn: controls *versus* LPS + STZ	0.049	0.049	0.0027
Dunn: LPS *versus* LPS + STZ	> 0.999	> 0.999	> 0.999

The T2 analyses were conducted using scalar T2 analysis and model-informed machine learning for T2 distribution to examine variations within the bone metaphyseal region across groups of mice with varying levels of inflammation (controls: control mice; LPS: mice with acute LPS-induced inflammation; LPS + STZ: LPS-injected and STZ-induced diabetic mice with the highest level of inflammation)

### MIML for T2 distribution of increasing levels of inflammation

**Fig. 4:**
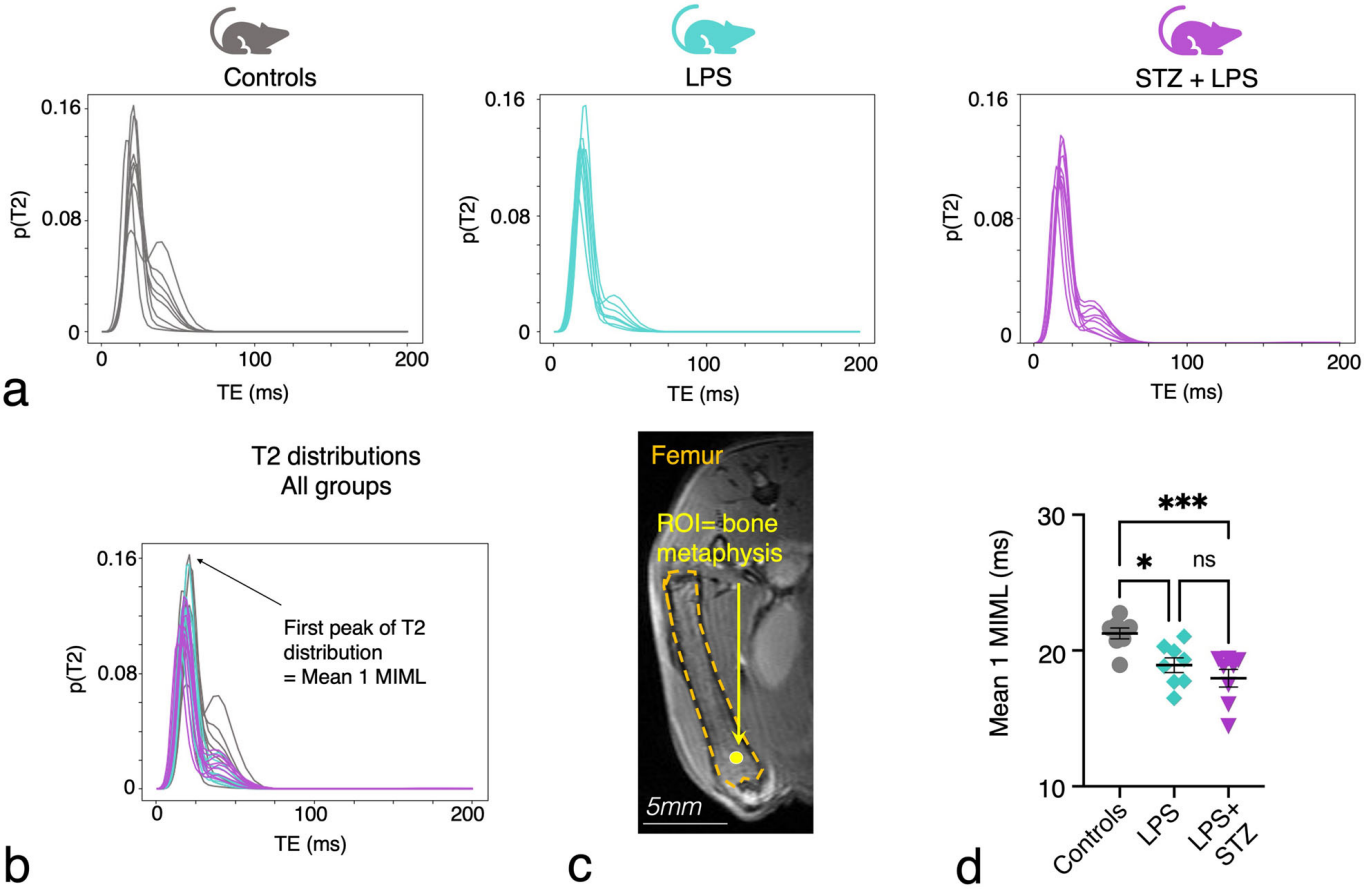
Deep learning-enhanced quantitative T2 microimaging in the inflamed femoral metaphysis. **a** Bone marrow T2 mapping in mice with increasing levels of inflammation (controls = control mice, LPS = mice with acute LPS-induced inflammation, STZ + LPS = mice with acute and diabetic inflammation), **b** T2 distribution of groups together. **c** Image defining the region of interest (ROI) within the femoral metaphysis. **d** The GMM mean parameter for the bone metaphysis, indicating the initial peak (mean 1) in the T2 distribution of femoral metaphysis as determined by model-informed machine learning (MIML) technique, across different groups. Each dot represents one mouse (*n* = 8 mice per group); Kruskal–Wallis test; *p < 0.05, **p < 0.01. TE, Echo time

In order to increase the sensitivity to discern microstructural changes in bone marrow structure with increasing levels of inflammation, we further analyzed the T2 distributions for the femoral bone marrow. By employing GMM in the distributions and plotting the means, variances, and weights, we sought to identify changes in the bone marrow T2 distributions among the experimental groups: controls, LPS, and STZ + LPS. The means of the first peak of the T2 distribution at the metaphyseal bone marrow was decreased with increasing levels of inflammation (Fig. 4a–c). The reduction in T2 relaxation time exhibited statistical significance according to Kruskal–Wallis analysis (Table 2). Notably, Dunn’s post-hoc analysis revealed significant differences in the comparison between controls and LPS mice, with an even more pronounced significance observed between controls and STZ + LPS mice (Fig. 4d and Table 2). However, the difference between LPS and STZ + LPS mice was not found to be statistically significant (Table 2).

### Iron sequestration within the inflamed marrow: correlation with neutrophil levels

**Fig. 5:**
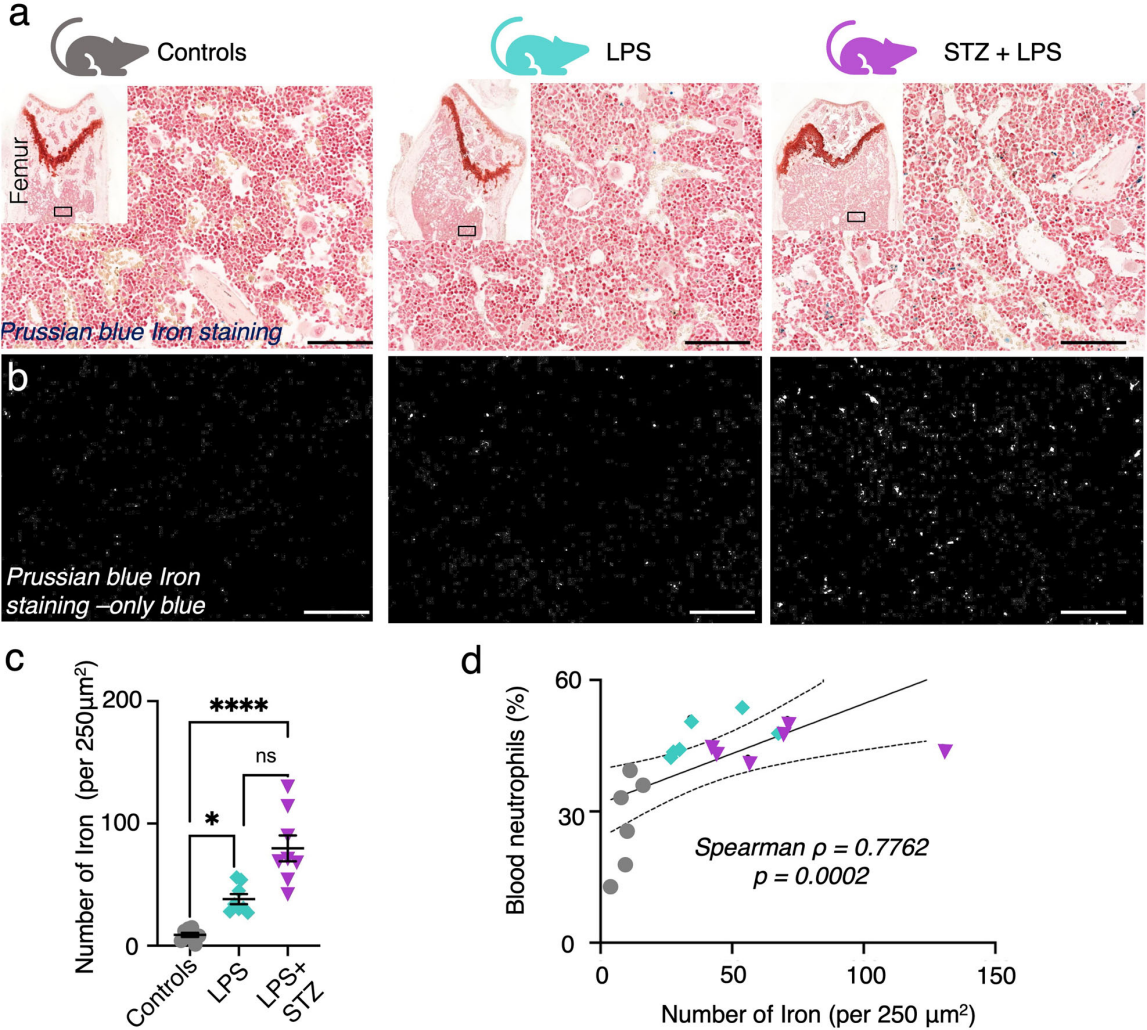
Bone marrow iron levels rise with increased inflammation. **a** Perls’ Prussian blue staining on 4 µm-sections of femoral bone marrow from controls, acute (LPS), and acute and diabetic inflammation (STZ + LPS) inflammation models. **b** Isolation of the blue channel from Perls’ Prussian blue stain to highlight iron levels. **c** Iron quantification in femoral bone marrow across a field-of-view measuring 250 × 250 µm^2^. **d** Correlation of T2 distribution of femoral metaphysis as determined by MIML mean 1 with histological iron quantification in the femoral bone marrow. Scale = 100 μm; each dot represents one mouse (*n* = 8 mice per group); Kruskal–Wallis test; **p < 0.05, **p < 0.0001)

Due to the association between iron content and low T2 values [46, 47], we hypothesized that the average of the initial peak within the T2 distribution could indicate iron sequestration within the bone marrow. Utilizing Prussian blue staining of histological slices from the femoral marrow (Fig. 5a), iron levels were quantified by thresholding the blue channel of the images (Fig. 5b), revealing elevated iron counts per ROI in mice with escalating levels of inflammation (Fig. 5c). Remarkably, a robust correlation was observed between blood neutrophil levels, indicative of inflammatory status, and iron count within the bone marrow (Spearman ρ = 0.776; *p* = < 0.001; Fig. 5d). Further examination of hematoxylin and eosin staining of the femoral metaphysis revealed no discernible differences between the experimental groups (Supplemental Fig. S1a). However, quantification of bone marrow cells from the femur of each group demonstrated significant changes (Supplemental Fig. S1b). This underscores the dynamic relationship between iron levels and inflammatory responses within the marrow microenvironment.

### Quantification of iron in the inflamed marrow: correlation with inflammation severity and T2 distribution values

**Fig. 6:**
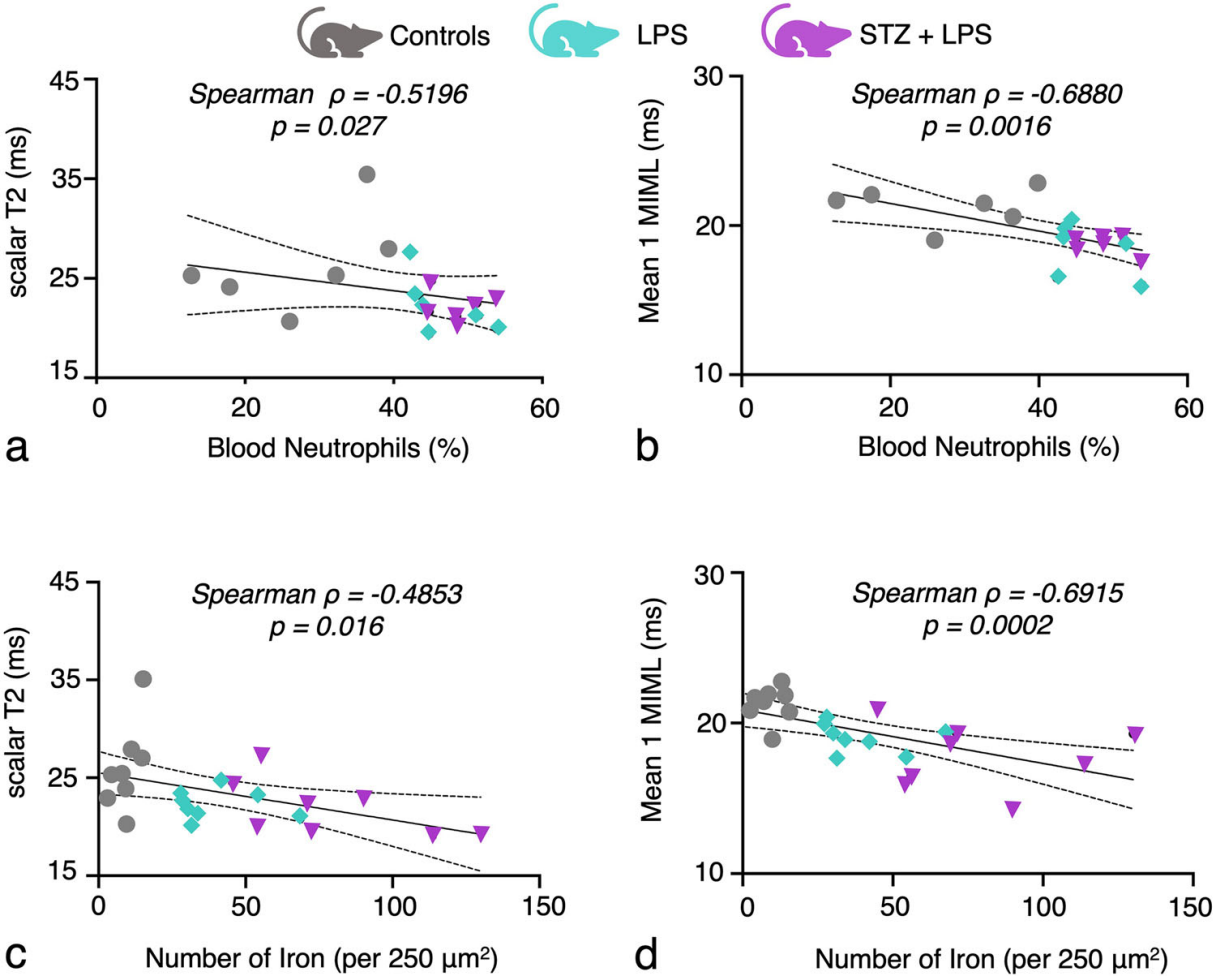
Correlation of blood neutrophils and bone marrow iron levels rise with scalar T2 and T2 distribution values during increasing inflammatory conditions. **a** Correlation of scalar T2 with blood neutrophil levels. **b** Correlation of T2 distribution of femoral metaphysis as determined by MIML mean 1 with blood neutrophil levels. **c** Correlation of scalar T2 values with histological iron quantification in the femoral bone marrow. **d** Correlation of T2 distribution as determined by MIML mean 1 with histological iron quantification in femoral bone marrow. MIML, Model-informed machine learning

Notably, scalar T2 values displayed a moderate correlation with blood neutrophil levels (Spearman ρ = -0.520; *p* = 0.027; Fig. 6a), while the first mean of the T2 distribution analysis exhibited a stronger correlation with neutrophil levels (Spearman ρ = -0.688; *p* = 0.002; Fig. 6b). This suggests a closer relationship between T2 distribution values and inflammatory responses compared to scalar T2 values. Moreover, considering the established correlation between a reduction in the first mean of the T2 distribution and iron deposition [27], correlation analysis revealed moderate associations between scalar T2 values and iron count (Spearman *r* = -0.485; *p* = 0.0162; Fig. 6c), whereas the first mean of the T2 distribution analysis showed a stronger correlation with iron count (Spearman ρ = -0.6915; *p* = < 0.001; Fig. 6e). These results emphasize the enhanced sensitivity of T2 distribution analysis in detecting subtle changes in bone marrow structure during inflammation, particularly in discerning alterations in iron values. Overall, the findings highlight the intricate interplay between T2 relaxation times, bone marrow composition, and inflammatory responses, underscoring the utility of T2 distribution analysis as a more sensitive approach for characterizing inflammatory processes within the marrow microenvironment.

## Discussion

This study demonstrates that T2-weighted imaging, scalar T2 mapping, and T2 distribution analysis are effective in detecting inflammation-induced changes in the femoral bone marrow in mice, particularly within the metaphysis, a critical site for hematopoietic activityT2-weighted imaging and scalar T2 mapping showed borderline significant decreases in SNR and T2 relaxation times, correlating with inflammation, while MIML-based T2 distribution analysis revealed more significant reductions in the first peak mean with increasing levels of inflammation. Additionally, histological analysis confirmed elevated iron sequestrations within the bone marrow, correlating strongly with both neutrophil levels and T2 distribution values. These findings underscore the complementary utility of qualitative and quantitative MRI techniques in assessing the bone marrow microenvironment and highlight T2 distribution analysis as a particularly sensitive method for characterizing inflammatory processes and iron sequestration during systemic inflammation.

This study elucidates a decrease in scalar T2 values of femoral bone marrow concurrent with escalating inflammatory levels, as indicated by rising blood neutrophil levels. Multiecho T2-weighted images were subjected to MIML analysis to enhance the accuracy and noise robustness of T2 distributions in the bone marrow during inflammation. Results revealed a reduction in the mean of the first peak of the T2 distribution within the metaphyseal bone marrow with increasing inflammation levels. The observed reduction in T2 scalar values and the first peak of T2 distribution values between LPS and STZ + LPS mice suggests that T2 values may be sensitive to the presence of STZ in addition to LPS. However, the lack of statistical significance limits the current applicability of this method in differentiating between these groups. This lack of significance could be due to the small sample size. Furthermore, it underscores the need for developing more precise methods for assessing T2 scalar values and the first peak of T2 distribution values.

To further correlate the altered T2 distribution values with inflammatory progression, Prussian blue staining of histological sections of the femur demonstrated elevated iron sequestration with increasing inflammation. A strong correlation was observed between bone marrow iron and blood neutrophil values, suggesting a direct association between heightened inflammation and increased iron absorption in the bone marrow. Importantly, the correlation of iron deposits and blood neutrophil levels with scalar T2 values was modest, whereas the correlation with T2 distribution values was notably high. This underscores the capability of T2 distribution analysis to discern microstructural iron deposits within the bone marrow accompanying increasing inflammation levels in a robust and accurate manner.

Our findings align with previous studies indicating that MRI can visualize major bone marrow pathologies [48, 49] and that T2 values correlate negatively with age [25]. MRI has proven to be a powerful tool for imaging the musculoskeletal system, offering exceptional soft tissue contrast for muscles, adipose tissue, tendons, and cartilage, while simultaneously providing valuable structural and functional insights [48, 49]. Although the metaphyseal region near the growth plate of long bones retains hematopoietically active red marrow in humans [16], the axial skeleton serves as the primary reservoir of active hematopoietic marrow in adulthood [15]. Therefore, translational studies should incorporate both axial and femoral marrow to account for potential regional variations in inflammatory responses. These compartments may display distinct quantitative and qualitative hematopoietic dynamics compared to murine femoral marrow. Such an approach would enable a more comprehensive understanding of inflammation-driven hematopoietic changes in human disease contexts.

The application of MIML for T2 distribution analysis provides deeper insights into tissue microstructure, leveraging its ability to resolve heterogeneity within each pixel beyond the capabilities of single-component T2 relaxometry. Building on prior work in brain imaging, where MIML was shown to differentiate tissue compartments effectively [28, 29], our study extends this approach to the bone marrow microenvironment. By analyzing the first peak in the T2 distribution, which correlates with short T2 values indicative of yellow bone marrow, fibrous tissue, and iron-rich regions, we propose that this peak reflects iron sequestration. This hypothesis aligns with the known influence of iron on T2 relaxation properties [46, 50, 51]. Additionally, the second peak in the T2 distribution likely represents red bone marrow and cartilage, underscoring the method’s ability to characterize diverse marrow compartments (Table 1).

To further investigate the observed decrease in scalar T2 and the means of the first T2 distribution peak with increased inflammation, we quantified iron levels in femoral bone marrow using Prussian blue staining on histological sections. As inflammation intensified—evidenced by elevated blood neutrophil levels—we observed a corresponding increase in iron sequestration within the inflamed bone marrow, reflecting the severity of the inflammatory response [46, 47]. These observations align with the physiological process of iron recirculation to the erythroid marrow during systemic inflammation. This redistribution supports heightened erythropoiesis, a hallmark of the body’s adaptive response to inflammatory states [50, 52, 53].

Studies in patients with elevated C-reactive protein levels, indicative of inflammation, have reported lower T2 (higher 1/T2 = R2) values, aligning with our observations [23]. Blood neutrophil levels and iron counts showed moderate correlations with scalar T2 values but stronger associations with T2 distribution values, highlighting T2 distribution analysis’s superior sensitivity for detecting inflammation-induced bone marrow changes. During inflammation, cytokines like interleukin 6 stimulate the overproduction of C-reactive protein and the hormone hepcidin, which regulates iron levels [23]. Hepcidin, a small peptide hormone released by hepatocytes, inhibits iron release into the bloodstream by binding to and deactivating the iron transporter ferroportin in cells such as duodenal enterocytes and tissue macrophages. This results in insufficient red blood cell production despite adequate or elevated iron stores in bone marrow macrophages, leading to a condition known as anemia of inflammation. Iron is stored as non-heme Fe3+ in molecules like ferritin and hemosiderin, which decreases T2* relaxation time, causing an increase in the R2* relaxation rate and decreased signals in the DWI of the bone marrow [23, 50, 52].

This study has several limitations. Firstly, our investigation was restricted to mouse models, and extending our research to human patients represents the next crucial step. Second, we exclusively utilized female mice of average age to minimize variation. Additionally, we faced challenges in visualizing the correlation between hematoxylin and eosin staining and cell number data with T2 measurements, particularly as lower cell numbers in tissues have been correlated with edema and higher T2 values [33]. Moreover, while we have focused on LPS-induced inflammation to model a bacteremia-like response [14], we recognize the importance of long-term monitoring of inflammatory status to visualize the resolution of inflammation, as well as tracking other models of inflammation, such as long-term viral infections and diseases such as metabolic syndrome and cancer. Future studies could incorporate these models to broaden our understanding of the bone marrow’s response to different inflammatory stimuli. Third, iron levels in the bone marrow can be affected by multiple factors, such as iron overload or deficiency. Our study focused exclusively on inflammatory variables, as iron metabolism remained stable within our mouse models. Future refinements that consider broader iron metabolism changes are necessary. Lastly, advances in biophysical modeling may reveal additional pertinent compartments requiring estimation.

It is increasingly recognized that the magnitude, persistence, and localization of inflammatory responses are crucial factors for prognosis and treatment monitoring. For instance, chronic low-grade inflammation, as seen in metabolic syndrome or atherosclerosis, often goes undetected by systemic blood biomarkers, which lack sensitivity to early, subclinical inflammation [1]. Moreover, there is growing interest in leveraging imaging technologies to monitor the efficacy of anti-inflammatory therapies. Treatments targeting the hematopoietic niche, such as anti-IL-1β therapies, could be evaluated in real-time using bone marrow imaging, enabling clinicians to tailor interventions based on early biological responses. While blood-based markers often provide a delayed and indirect measure of inflammation, direct imaging of the bone marrow offers the opportunity to capture immune activity at its source.

Our study introduced a novel, noninvasive MRI-based approach to visualize critical microenvironmental changes, including vascular remodeling, iron sequestration, and shifts in marrow composition, typically undetectable through blood tests. This study provides evidence of a decrease in scalar T2 values within the femoral bone marrow in tandem with rising levels of inflammation, as displayed by elevated blood neutrophil counts. Leveraging multi-echo T2 MRI combined with MIML analysis, we elucidated even more pronounced changes in T2 distributions, Histological examination using Prussian Blue staining corroborated these findings. Notably, while the correlation of iron levels and blood neutrophil levels with scalar T2 values was moderate, the association with T2 distribution values was notably stronger, emphasizing the superior sensitivity of T2 distribution analysis in discerning microstructural changes accompanying inflammation of the marrow. These findings highlight the specificity of bone marrow imaging in reflecting immune activity, after increasing levels of inflammation, thereby offering an early biomarker for acute and “silent” chronic inflammation, enabling timely intervention strategies. This capability has profound implications for patient stratification, treatment monitoring, and tracking disease progression.

## Supplementary information

**Additional file 1: Figure S1.** Bone marrow H&E staining does not show changes, yet a reduction of a number of bone marrow cells with increasing inflammatory levels. (A) H&E staining on 4μm sections of femoral bone marrow from control (CTR), acute (LPS), and diabetic and acute inflammation (STZ+ LPS) mice. (B) Quantification of the number of isolated bone marrow cells per femur. (scale = 100 μm; Each dot represents one mouse (*n* = 5 mice per group); Kruskal–Wallis test; ns non-significant, **p < 0.01).

## Data Availability

The data is available upon reasonable request.

## Abbreviations

[18F]FDG-PET: Fluorodeoxyglucose positron emission tomography
DWI: Diffusion-weighted imaging
GMM: Gaussian mixture model
LPS: Lipopolysaccharide
MIML: Model-informed machine learning
RARE: Rapid acquisition with refocused echoes
ROI: Region-of-interest
SNR: Signal-to-noise ratio
STZ: Streptozotocin
TE: Echo time
TR: Repetition time
