# Supplementary material for: Improved MRI detection of inflammation-induced changes in bone marrow microstructure in mice: a machine learning-enhanced T2 distribution analysis
Source: Eur Radiol Exp. 2025 Mar 26;9:39. doi: 10.1186/s41747-025-00574-1 (PMC11947359; doi:10.1186/s41747-025-00574-1)
Supplement: Supplementary file 1 — Additional file 1: Figure S1. Bone marrow H&E staining does not show changes, yet a reduction of a number of bone marrow cells with increasing inflammatory levels. (A) H&E staining on 4μm sections of femoral bone marrow from control (CTR), acute (LPS), and diabetic and acute inflammation (STZ+ LPS) mice. (B) Quantification of the number of isolated bone marrow cells per femur. (scale = 100 μm; Each dot represents one mouse (n = 5 mice per group); Kruskal–Wallis test; ns non-significant, **p < 0.01). [file 41747_2025_574_MOESM1_ESM.pdf]

Improved MRI detection of inflammation-induced changes in bone marrow microstructure in mice: a machine learning-enhanced T2 distribution analysis

ELECTRONIC SUPPLEMENTARY MATERIAL

Methods

Histology. For comparing the findings in MR with a ground truth, histological exams were performed on the murine bone marrow of left femur. Femur sections of the inflammatory groups were stained with hematoxyline and eosin (H&E).

Cell count bone marrow cells. To obtain the number of bone marrow cells per femur, mice from all groups (n=5) were sacrificed, and bone marrow cells were flushed with DMEM from the femur and tibia and isolated through a 70-μm mesh. After osmotic erythrocyte lysis, cells were pelleted, counted. Counting the number of unstained (viable) cells was done after incubation with Trypan Blue using a hemocytometer.

__Supplemental Figure__

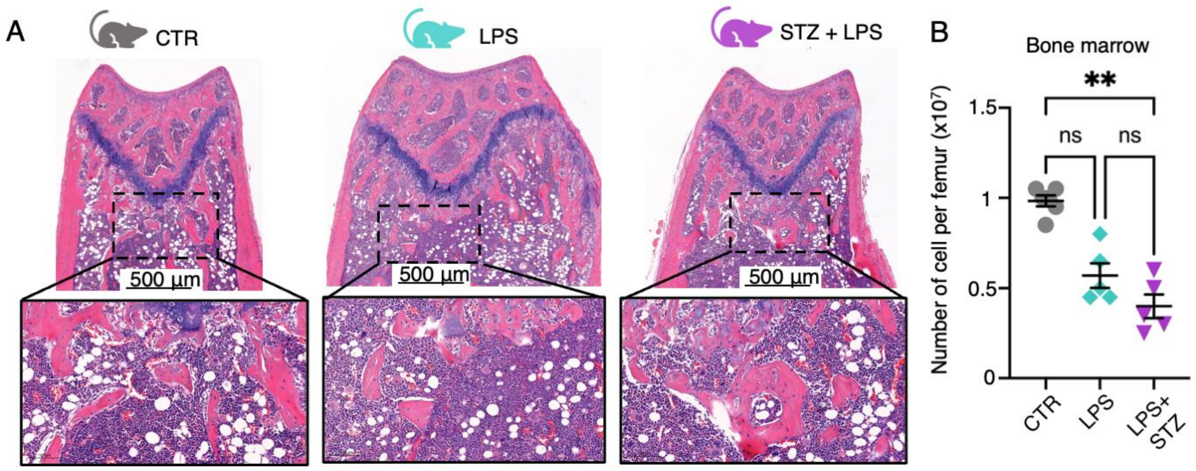

Figure S1 | Bone marrow H&E staining does not show changes, yet reduction of number of bone marrow cells with increasing inflammatory levels. (A) H&E staining on 4µm sections of femoral bone marrow from control (CTR), acute (LPS), and diabetic and acute inflammation (STZ+ LPS) mice. (B) Quantification of the number of isolated bone marrow cells per femur. (scale = 100µm; Each dot represents one mouse (n=5 mice per group); Kruskal-Wallis test; ns= non-significant, **P < 0.01)
